# Ectodysplasin A2 receptor-NF-κB-inducing kinase axis: a new player in muscle wasting to cancer cachexia

**DOI:** 10.1038/s41392-023-01617-y

**Published:** 2023-10-09

**Authors:** Janusz von Renesse, Peter Mirtschink

**Affiliations:** 1 Department of Visceral, Thoracic and Vascular Surgery, University Hospital and Faculty of Medicine Dresden, Technische Universität Dresden, Dresden, Germany; 2 National Center for Tumor Diseases (NCT), Partner Site Dresden, Dresden, German Cancer Research Center (DKFZ), Heidelberg, Germany; 3 Institute for Clinical Chemistry and Laboratory Medicine, University Hospital and Faculty of Medicine, Technische Universität Dresden, Dresden, Germany

**Keywords:** Predictive markers, Oncogenesis

In a recent study published in *Nature*, Bilgic et al. have elucidated a crucial role of Ectodysplasin A2 receptor (EDA2R) and its downstream effector, NF-κB-inducing kinase (NIK), in the pathogenesis of cancer cachexia. This discovery suggests interventional potential to ameliorate muscle loss by targeting EDA2R signaling.^1^

Cancer cachexia is a complex syndrome characterized by weight loss, muscle atrophy, and metabolic dysregulation, impeding cancer treatment and reducing patients’ quality of life. Cachexia can be seen as end-stage systemic manifestation common to various chronic diseases. Distinct triggers like aging, COPD, HIV, and cancer result in unresolved metabolic or inflammatory stress, leading to a maladaptive state.^2^

Cachexia’s clinical signs, like physical frailty, stem from accelerated protein degradation, especially in skeletal muscle. Notably, despite the variety of initiating factors, substantial commonality exists in the downstream pathophysiological mechanisms underpinning cachexia across different conditions.^2^ For instance, cytokines - often released by immune or tumor cells - facilitate muscle wasting and weight loss. Most consistently upregulated cytokines in cachexia are tumor necrosis factor-alpha (TNF-α), IL-6, IL-1, and Tweak.^3^ For example, TNF-α, first known as cachexin, boosts skeletal muscle degradation through the ubiquitin-proteasome pathway and autophagy upregulation.

A common downstream effect of such inflammation-induced catabolic activity is the activation of the NF-κB pathway. As a critical regulator of immune and inflammatory responses, this pathway can be stimulated by TNF-α and IL-1β. When activated in skeletal muscle, the NF-κB pathway precipitates proteasomal protein degradation. In adipose tissue, it promotes triglyceride breakdown and restricts their uptake by downregulating hormone-sensitive lipase.^3^

In the presented study the authors noted an upregulation of EDA2R expression in muscle tissue of cachectic mice in a lung carcinoma and melanoma model. Intriguingly, a similar upregulation was observed in muscle biopsies from patients with cachexia due to pancreatic and colorectal cancer.^1^ This observation not only points to a potential role for EDA2R-mediated muscle loss across different types of malignancies but also suggests the possibility of translational applications.

**Fig. 1:**
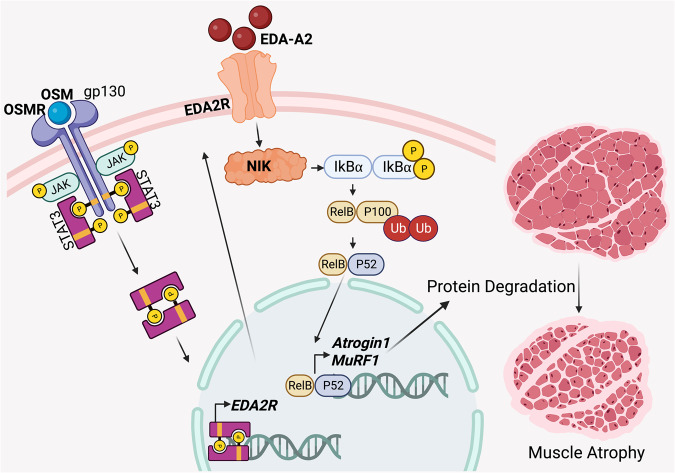
Elevated levels of Oncostatin M (OSM) observed in tumor-bearing mice and cachectic cancer patients induce an increased expression of the transmembrane receptor for the A2 isoform of Ectodysplasin A (EDA2R), presumably via the JAK/STAT3 pathway. Subsequently stimulation of EDA2R by its ligand EDA-A2 activates the noncanonical NF-κB pathway in a NIK kinase dependent manner inducing the expression of Atrogin1 and the muscle RING-finger protein-1 (MuRF1). This process results in muscle wasting due to accelerated protein degradation. Cartoon created with Biorender.com

The researchers demonstrate that EDA2R-NIK signaling is integral to tumor-induced muscle wasting through activation of the non-canonical NF-κB pathway (Fig. 1). EDA2R knockout (EDA2R-KO) and skeletal muscle-specific NIK knockout (Myo-NIK-KO) mice displayed resistance to tumor-induced muscle loss compared to their wild-type counterparts.^1^

Indeed, a critical aspect of the EDA2R-NIK signaling pathway is its regulation of muscle-specific ubiquitin ligases, Atrogin1 and MuRF1, which are known to play key roles in muscle atrophy.^2^ Interestingly, in EDA2R-KO, and Myo-NIK-KO mice, the tumor-induced upregulation of Atrogin1 and MuRF1 was significantly diminished, further underscoring the role of these signaling pathways in muscle wasting.

The study further investigates the mechanism, by which tumors upregulate EDA2R expression in muscle tissue. It is demonstrated that the cytokine oncostatin M (OSM), typically secreted by tumors, is capable of enhancing EDA2R expression. In tandem, OSM and EDA-A2 (EDA2R’s ligand) amplify atrophy-related gene expression and exacerbate muscle wasting.

The researchers also highlight the importance of the OSM receptor (OSMR) in this process. Skeletal muscle-specific OSMR knockout (Myo-OSMR-KO) mice were largely protected from weight loss and muscle wasting, which emphasizes the role of OSM-OSMR signaling in activating EDA2R-NIK signaling and promoting muscle atrophy.

Importantly, OSM was found to be elevated in the blood plasma of Lewis Lung Carcinoma (LLC) tumor-bearing mice.^1^ Furthermore, the upregulation of EDA2R by OSM could also be reproduced in vitro by treating primary myotubes with OSM. This raises questions about the source of OSM. Is it the tumor itself, a systemic inflammatory response, or both?

In exploring shared molecular mechanisms, we might draw further insights from conditions associated with muscle deprivation, such as obesity, diabetes, and aging. Interestingly, a recent preprint highlighted EDA2R expression to be strikingly well correlated to the increasing age of donors in a tissue-type independent manner.^4^ Building on these observations, the findings of the presented study suggest that tumors may exploit and accelerate the intrinsic pathomechanisms of age-related muscle degradation. This raises an intriguing question: Why do tumors do this? It could be a bystander effect, indirectly triggered by systemic inflammatory responses, but it could also be a targeted strategy, e.g. by tumor-driven reprogramming of hematopoietic stem/progenitor cells (HSPCs). A tumor-driven reprogramming of HSPCs might consequently lead to an increased production of OSM in leukocytes, subsequently promoting muscle loss. Furthermore, the activation of EDA2R may have a significant impact on metabolite release through muscle protein breakdown. Could EDA2R activation drive the production of specific metabolites that, in turn, facilitate tumor growth? This is a concept echoed in other muscle-wasting conditions such as diabetes, where muscle degradation is activated to release metabolites, like alanine, that aid gluconeogenesis. Addressing these follow-up questions could not only contribute to our mechanistic understanding of muscle loss but also potentially provide a wider perspective by tying the study’s findings into the broader context of metabolic disorders. Comparatively, the failure of current anti-inflammatory medications against cachexia, might be more an issue of specificity rather than an argument against this overarching concept.^2^ The involvement of the non-canonical NF-κB pathway activation and the specific involvement of OSM as secreted EDA2R inducer in the presented study provide a beacon of hope toward a more specific understanding of muscle loss and novel therapeutic approaches.

Currently, the most effective strategy for patients at risk remains prevention. Regrettably, cachexia is often undetected until it has progressed too far for successful interventions to reverse muscle loss.^2^ Potential early warning markers, like tumor-derived OSM, often lack specificity. Looking at cachexia as a maladaptive response, the body’s attempt to mobilize energy might result in the release of metabolites that could signify the early onset of breakdown well before any clinical manifestation. Hence a highly standardized quantitative measurement of metabolic markers of muscle decline in patient’s plasma or urine based on Liquid-Chromatography coupled Tandem-Mass spectrometry (LC-MS/MS) or, due to its straightforward sample preparation, Nuclear Magnetic Resonance (NMR) could prove invaluable in detecting muscle wasting over time.

Given the complexity of metabolic processes involved, interventions might have unexpected and potentially opposing effects on disease development. A recent preclinical study demonstrated this intricacy by showing that a ketogenic diet slowed tumor growth but accelerated cachexia, ultimately hastening death.^5^ Consequently, the analysis of such metabolic profiles could serve as a crucial tool not only for improved risk stratification and pharmacological decision-making but also for monitoring the impact of both pharmacological and metabolic interventions, ensuring they do not inadvertently worsen cachexia or tumor progression.
